# Staphylococcus aureus Responds to Physiologically Relevant Temperature Changes by Altering Its Global Transcript and Protein Profile

**DOI:** 10.1128/mSphere.01303-20

**Published:** 2021-03-17

**Authors:** Raeven A. Bastock, Emily C. Marino, Richard E. Wiemels, Donald L. Holzschu, Rebecca A. Keogh, Rachel L. Zapf, Erin R. Murphy, Ronan K. Carroll

**Affiliations:** a Department of Biological Sciences, Ohio University, Athens, Ohio, USA; b Molecular and Cellular Biology Program, Ohio University, Athens, Ohio, USA; c Infection and Tropical Disease Institute, Ohio University, Athens, Ohio, USA; d Honors Tutorial College, Ohio University, Athens, Ohio, USA; e Heritage College of Osteopathic Medicine, Department of Biomedical Sciences, Ohio University, Athens, Ohio, USA; University of Iowa

**Keywords:** *Staphylococcus aureus*, colonization, host cell invasion, posttranscriptional control mechanisms, regulation, temperature

## Abstract

Staphylococcus aureus is an opportunistic pathogen that colonizes the anterior nares of 30 to 50% of the population. Colonization is most often asymptomatic; however, self-inoculation can give rise to potentially fatal infections of the deeper tissues and blood. Like all bacteria, S. aureus can sense and respond to environmental cues and modify gene expression to adapt to specific environmental conditions. The transition of S. aureus from the nares to the deeper tissues and blood is accompanied by changes in environmental conditions, such as nutrient availability, pH, and temperature. In this study, we perform transcriptomics and proteomics on S. aureus cultures growing at three physiologically relevant temperatures, 34°C (nares), 37°C (body), and 40°C (pyrexia), to determine if small scale, biologically meaningful alterations in temperature impact S. aureus gene expression. Results show that small but definite temperature changes elicit a large-scale restructuring of the S. aureus transcriptome and proteome in a manner that, most often, inversely correlates with increasing temperature. We also provide evidence that a large majority of these changes are modulated at the posttranscriptional level, possibly by sRNA regulatory elements. Phenotypic analyses were also performed to demonstrate that these changes have physiological relevance. Finally, we investigate the impact of temperature-dependent alterations in gene expression on S. aureus pathogenesis and demonstrate decreased intracellular invasion of S. aureus grown at 34°C. Collectively, our results demonstrate that small but biologically meaningful alterations in temperature influence S. aureus gene expression, a process that is likely a major contributor to the transition from a commensal to pathogen.

**IMPORTANCE** Enteric bacterial pathogens, like Escherichia coli, are known to experience large temperature differences as they are transmitted through the fecal oral route. This change in temperature has been demonstrated to influence bacterial gene expression and facilitate infection. Staphylococcus aureus is a human-associated pathogen that can live as a commensal on the skin and nares or cause invasive infections of the deeper tissues and blood. Factors influencing S. aureus nasal colonization are not fully understood; however, individuals colonized with S. aureus are at increased risk of invasive infections through self-inoculation. The transition of S. aureus from the nose (colonization) to the body (infection) is accompanied by a modest but definite temperature increase, from 34°C to 37°C. In this study, we investigate whether these host-associated small temperature changes can influence S. aureus gene expression. Results show widespread changes in the bacterial transcriptome and proteome at three physiologically relevant temperatures (34°C, 37°C, and 40°C).

## INTRODUCTION

Staphylococcus aureus is a Gram-positive bacterium that has a both commensal and highly pathogenic relationship with the human host (1). As a commensal, S. aureus colonizes the anterior nares in approximately 30% of the global population (2, 3). While colonization of the anterior nares is most commonly asymptomatic, it is also associated with a higher risk of invasive infections (4). As a pathogen, S. aureus can cause a multitude of diseases throughout the human body that range from mild infections of the skin and soft tissues to life-threatening conditions such as necrotizing fasciitis, toxic shock syndrome, or septicemia (1). Previous studies have shown that self-inoculation with the colonizing strain often leads to invasive S. aureus infections (4–7). Despite this, very little is known about the physiological changes and genetic factors that influence S. aureus transition from colonization to invasion states, when the bacteria transition from the nares to an internal body site.

Regulation of gene expression in response to environmental stimuli is a well-documented phenomenon in bacteria. Bacteria use a variety of mechanisms, at both the transcriptional and posttranscriptional levels, to alter gene expression in response to specific environmental stimuli. Environmental cues known to influence bacterial gene expression include, but are not limited to, nutrient availability, osmotic pressure, pH, and temperature. Surprisingly, while gene regulation in response to numerous environmental cues has been studied in S. aureus (8–12), few have investigated whether this pathogen utilizes temperature as a signal to modulate gene expression, and to our knowledge, none have investigated this relatively narrow temperature range (13–18). Based on the bimodal lifestyle of S. aureus (as both a commensal and a pathogen), this study tests the hypothesis that the temperature upshift experienced when transitioning from the nares (34°C) (19) to inside the human body (37°C) is a biologically relevant signal that influences gene expression and, potentially, pathogenesis. Specifically, it is hypothesized that growth of S. aureus at the temperatures associated with colonization and invasive infection leads to distinct transcriptomic and proteomic profiles that facilitate adaptation of the bacteria to the environment associated with each temperature.

To test these hypotheses, the global response of S. aureus to physiologically relevant temperatures of 34°C (nares), 37°C (internal body), and 40°C (extreme pyrexia) was examined using parallel proteomic and transcriptomic analyses. Findings demonstrate that growth of S. aureus at 34°C, 37°C, and 40°C results in distinct transcript and protein profiles. Comparison of the transcriptomic and proteomic data sets generated at each temperature identified distinct sets of genes that are subject to temperature-dependent transcriptional or posttranscriptional regulation. Interestingly, the greatest variation in the S. aureus transcriptome and proteome is seen when comparisons are made between profiles generated at 34°C and those generated at either 37°C or 40°C. These data suggest that environmental temperature may play a major role in signaling and adaptation of S. aureus during colonization of the nares and during transition to warmer environments of the human body. Phenotypic analyses were used to confirm and validate the proteomic differences observed for selected virulence factors at the three temperatures tested. Finally, significant variations in eukaryotic cell invasion by S. aureus at 34°C and 37°C demonstrate the physiological relevance of temperature-dependent gene regulation in this important virulence-associated process.

## RESULTS

### S. aureus growth at different physiologically relevant temperatures.

**FIG 1:**
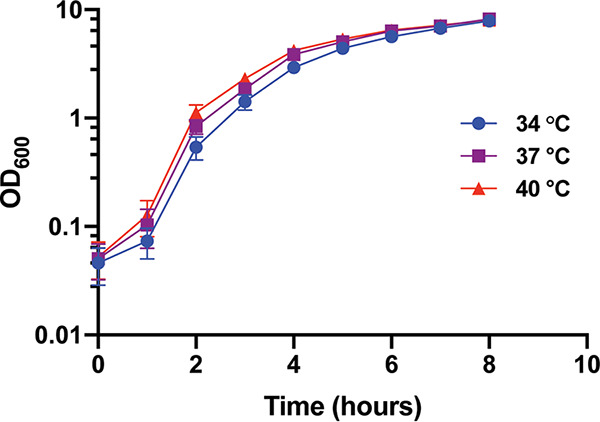
Growth of S. aureus at physiologically relevant temperatures. S. aureus cultures were grown in TSB at the indicated temperature and growth monitored by OD_600_. Cultures grown at 34°C displayed a prolonged lag phase compared to cultures grown at 37°C and 40°C. Cultures were grown in triplicate.

The goal of this study was to examine how temperature influences gene expression and protein production in S. aureus at three temperatures: 34°C, 37°C, and 40°C. As a prelude to this study, the influence of these temperatures on S. aureus growth was examined. Triplicate cultures of wild-type S. aureus (USA300 strain AH1263) were incubated at the three temperatures and growth was monitored over an 8 h period by measurement of the optical density at 600 nm (OD_600_) (Fig. 1). Cultures grown at 34°C display a slight growth defect compared to those at 37°C and 40°C. At 34°C, the culture experiences a prolonged lag phase compared to that of cultures at 37°C and 40°C. Of note, S. aureus cultures reach the same OD_600_ in stationary phase at all temperatures tested. Although the difference in lag phase was modest, cultures used for all subsequent experiments were normalized to the same OD_600_ as a means to account for altered growth of S. aureus at various temperatures.

### The S. aureus transcriptome is altered by environmental temperature.

Transcriptome sequencing (RNAseq) analyses were performed to quantitatively analyze alterations in transcript levels following the growth of S. aureus at the three different temperatures. Total RNA was isolated from wild-type S. aureus following growth to the postexponential phase at each temperature (corresponding to 6 h growth at 37°C). Using the RNAseq data generated (in triplicate at each temperature), two differential expression analyses (DEAs) were performed comparing transcript levels at 34°C versus 37°C and 37°C versus 40°C. Transcripts exhibiting a fold change ≥ 2 were considered for further analysis. In total, 87 transcripts showed variation in expression levels between 34°C versus 37°C, with 67 transcripts having an increase in abundance at 34°C and 20 having an increase at 37°C. The 37°C versus 40°C DEA revealed 60 transcripts with altered expression, with 47 transcripts increased at 37°C and 13 increased at 40°C. Collectively, these data suggest that as S. aureus transitions from the nares (34°C) to internal body sites (37 to 40°C), alterations in transcript expression result in a large number of transcripts being downregulated and a smaller number being upregulated.

**FIG 2:**
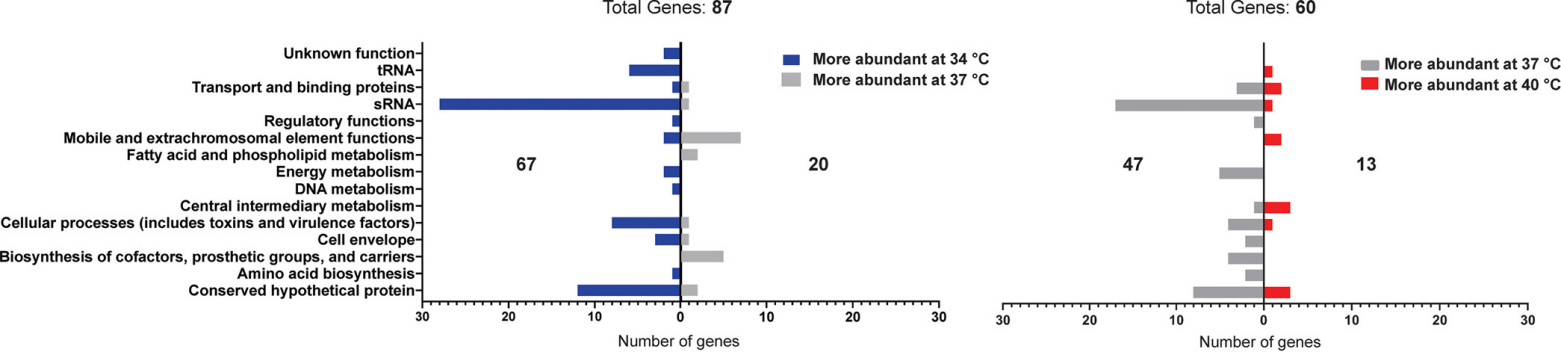
Differential gene expression analysis of S. aureus at 34°C versus 37°C and 37°C versus 40°C. Genes showing a 2-fold or greater difference in expression were grouped into functional clusters and plotted based on the temperature at which their expression was greater. 87 transcripts show altered expression between 34°C and 37°C, with 67 having higher levels at 34°C. 60 transcripts show altered abundances between 37°C and 40°C, with 47 having higher levels at 37°C.

To investigate which cellular processes are influenced by temperature-dependent alterations in gene expression, transcripts showing variation in abundance (by DEA analysis above) were placed into functional categories based on their annotations in the COG database (20) and grouped based on increased or decreased abundance in each of the DEAs (Fig. 2). Interestingly, the functional cluster with the largest number of altered transcripts in both analyses is sRNAs. Also in both analyses, the majority of sRNAs affected show increased abundance at the lower temperature in comparison (28 of 29 are more abundant at 34°C versus 37°C, while 17 of 18 are more abundant at 37°C versus 40°C). Furthermore, 15 sRNA transcripts show a ≥2-fold increase in abundance in both analyses (Table 1). While these data suggest that expression of sRNAs in S. aureus is strongly influenced by temperature, it is also possible that the stability of these sRNAs could be temperature dependent, albeit in a manner that correlates inversely with temperature. Regardless of the underlying mechanism, the data clearly demonstrate that sRNA levels in S. aureus are influenced by small, defined changes in environmental temperature.

**TABLE 1:** sRNAs with a ≥2-fold change in each comparison

USA300 no.	Name	34°C expression value^*a*^	37°C expression value^*a*^	40°C expression value^*a*^	Ratio 34°C/37°C	Ratio 37°C/40°C	Ratio 34°C/40°C
SAUSA300s031	sprD	167.73	31.73	3.88	5.29	8.18	43.23
SAUSA300s050	RsaD	5,359.55	2,090.61	86.34	2.56	24.21	62.07
SAUSA300s056	RsaK	159.26	31.86	14.36	5.00	2.22	11.09
SAUSA300s089	Teg49	915.4	87.65	8.88	10.44	9.87	103.09
SAUSA300s104	Teg141	73.05	27.92	8.87	2.62	3.15	8.24
SAUSA300s148	Teg23	3,776.04	1,670.10	543.96	2.26	3.07	6.94
SAUSA300s153	Teg16	1,059.05	248.94	60.67	4.25	4.10	17.46
SAUSA300s171	Sau-6569	115.92	48.8	16.85	2.38	2.90	6.88
SAUSA300s220	sRNA140	43.13	18.32	1.54	2.35	11.90	28.01
SAUSA300s227	sRNA178	429.91	215.46	98.35	2.00	2.19	4.37
SAUSA300s233	sRNA205	210.64	85.17	25.82	2.47	3.30	8.16
SAUSA300s239	sRNA260	31.39	10.54	1.2	2.98	8.78	26.16
SAUSA300s261	sRNA397	79.21	33.24	8.48	2.38	3.92	9.34
SAUSA300s274	tsr10	65.87	26.26	8.6	2.51	3.05	7.66
SAUSA300s282	tsr18	519.56	254.9	16.71	2.04	15.25	31.09

a Average expression value in RPKM (reads per kilobase per million mapped reads).

With the exception of conserved hypothetical proteins, the functional cluster with the next largest number of altered genes was “cellular processes,” a group that includes genes encoding toxins and other virulence factors. As was the case for sRNAs, most genes in this category showed increased abundance at lower temperatures (8 of 9 were more abundant at 34°C versus 37°C, while 4 of 5 were more abundant at 37°C versus 40°C). Two genes (*sak* and *sbi*) showed ≥2-fold variation in both analyses, resulting in a total of 12 genes that showed ≥2-fold variation in transcript level in one or both analysis (34°C versus 37°C or 37°C versus 40°C) (Table 2). While most of these cellular processes transcripts had increased abundance at lower temperatures, notably, the αPSM transcript and *dps* displayed increased abundance at higher temperatures. Thus, while several genes encoding virulence factors show temperature-dependent regulation in S. aureus, the correlation between temperature and expression level can be either direct (expression level increases with increased temperature) or inverse (expression level decreases with increased temperature).

**TABLE 2:** Temperature-regulated virulence genes

USA300 no.	Name	34°C expression value^*a*^	37°C expression value^*a*^	40°C expression value^*a*^	Ratio 34°C/37°C	Ratio 37°C/40°C	Ratio 34°C/40°C
SAUSA300_2572	*aur*	92.1	25.1	16.52	3.67	1.52	5.58
SAUSA300_0949	*sspC*	857.27	240.1	172.27	3.57	1.39	4.98
SAUSA300_1975	*lukA*	102.95	34.46	21.72	2.99	1.59	4.74
SAUSA300_0951	*sspA*	974.25	330.7	224.12	2.95	1.48	4.35
SAUSA300_0950	*sspB*	848.61	291.67	173.84	2.91	1.68	4.88
SAUSA300_1922	*sak*	292.07	111.8	50.36	2.61	2.22	5.80
SAUSA300_1974	*lukB*	129.98	52.93	35.3	2.46	1.50	3.68
SAUSA300_2364	*sbi*	360.11	175.06	84.49	2.06	2.07	4.26
SAUSA300_1990	*agrD*	1,322.9	774.75	308.9	1.71	2.51	4.28
SAUSA300_0278	*esxA*	6,275	5,439	2,544.2	1.15	2.14	2.47
SAUSA300_2092	*dps*	897.51	1,028.9	2,887	0.87	0.36	0.31
SAUSA300_0424.1	*PSMa*	14.24	32.2	53.56	0.44	0.60	0.27

a Average expression value in RPKM (reads per kilobase per million mapped reads).

### The cytoplasmic proteome of S. aureus is minimally affected by growth at different temperatures.

**FIG 3:**
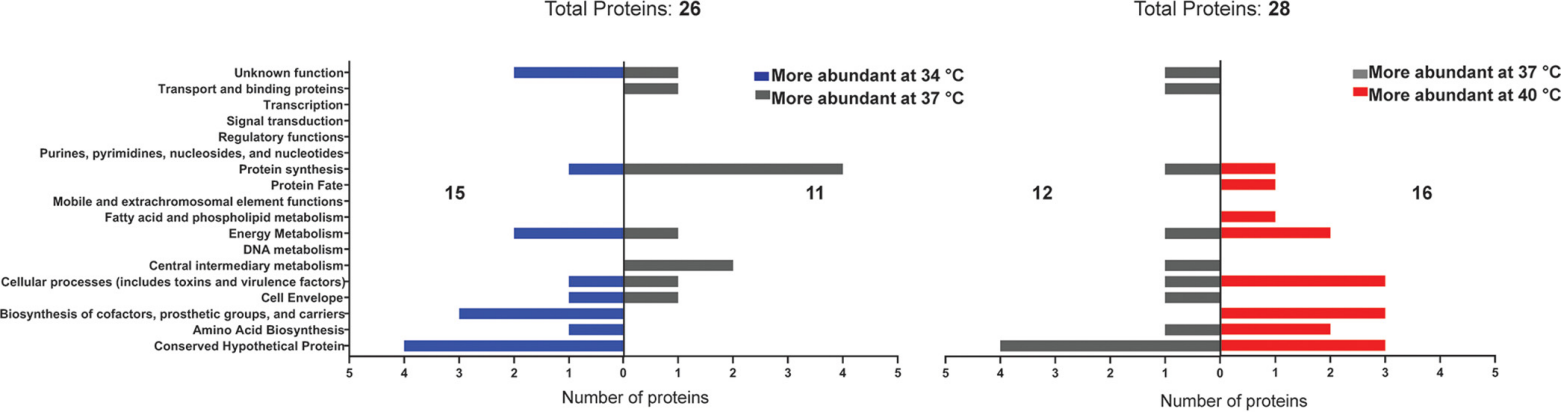
Differential abundance of S. aureus cytoplasmic proteins at 34°C versus 37°C and 37°C versus 40°C. Proteins showing a 2-fold or greater difference in expression were grouped into functional clusters and plotted based on the temperature at which their expression was greater. 26 proteins show altered abundance between 34°C and 37°C, with 15 of those demonstrating higher abundance at 34°C. 28 proteins show altered abundances between 37°C and 40°C, with 12 of those demonstrating higher abundance at 37°C.

Protein levels do not necessarily correlate with transcript levels. Therefore, in parallel with the transcriptomic analysis above, the S. aureus proteome was also examined. To identify differences in intracellular protein levels, mass spectrometry was performed on cell lysates from the same S. aureus cultures described for the RNAseq analysis. Incubation temperatures were again paired for analysis (as outlined above for DEAs), and proteins displaying a minimum of a 2-fold change were clustered by function to identify common pathways altered by temperature. Overall, the data show that the intracellular proteome of S. aureus is relatively unchanged by growth temperature, with only 26 proteins showing a ≥2-fold change in abundance between 34°C and 37°C and 28 showing a ≥2-fold change in abundance between 37°C and 40°C (Fig. 3 and see Data Set S2 in the supplemental material). Comparison of 34°C and 37°C reveals that 15 proteins show increased expression at 34°C, while 11 proteins show increased abundance at 37°C (Fig. 3). Comparison of cultures at 37°C and 40°C show 12 upregulated proteins at 37°C and 16 that are upregulated at 40°C. While the overall number of altered proteins was low, some proteins did exhibit expected changes. For example, the cold shock protein CspC showed an increased abundance at 34°C compared to that at 37°C (3.14-fold). Heat shock and chaperone proteins GroEL/ES, DnaK, CtsR, and ClpB did not show a ≥2 -fold change between 37°C versus 40°C but, nonetheless, were found in slightly greater abundances at 40°C (1.47-fold, 1.43-fold, 1.33-fold, 1.58-fold, and 1.18-fold, respectively) (Data Set S2). Taken together, these expression patterns suggest that temperature differences are being sensed. While no single functional cluster displays large alterations, many proteins observed in the cytoplasmic DEAs are involved in cellular metabolism.

10.1128/mSphere.01303-20.2 DATA SET S2 Cytoplasmic proteomics data analysis. Download Data Set S2, XLSX file, 0.08 MB. Copyright © 2021 Bastock et al. 2021 Bastock et al. https://creativecommons.org/licenses/by/4.0/ This content is distributed under the terms of the Creative Commons Attribution 4.0 International license.

### Temperature causes large-scale alterations in the S. aureus secretome.

**FIG 4:**
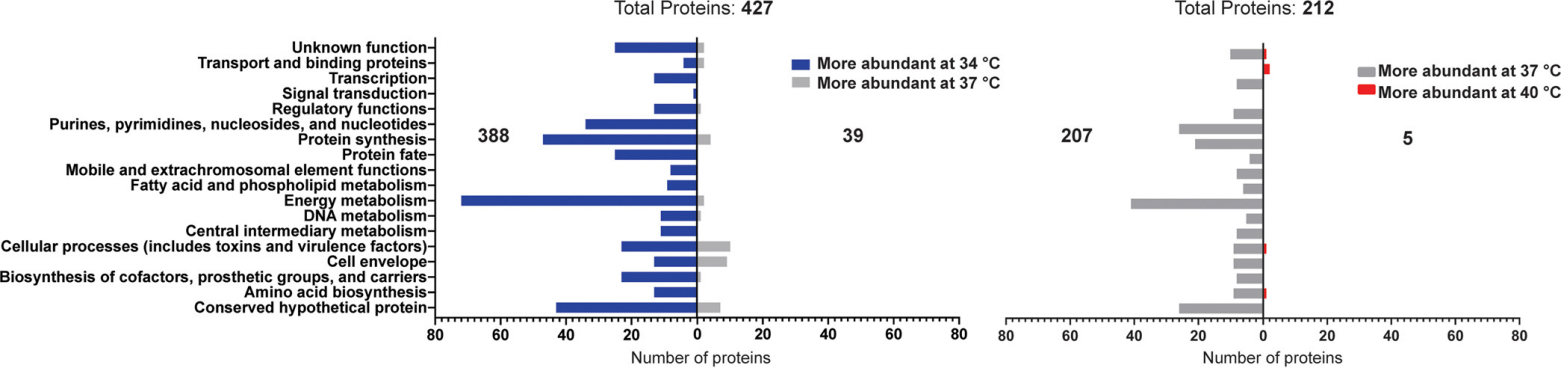
Differential abundance of S. aureus secreted proteins at 34°C versus 37°C and 37°C versus 40°C. Proteins showing a 2-fold or greater difference in expression were grouped into functional clusters and plotted based on the temperature at which their expression was greater. 427 proteins show altered abundance between 34°C and 37°C, with 388 of those showing higher abundance at 34°C. 212 proteins show altered abundances between 37°C and 40°C, with 207 of those showing higher abundance at 37°C.

The secreted proteome of S. aureus is imperative for the establishment of an infection and survival of the bacteria *in vivo*; thus, the impact of environmental temperature on the S. aureus secretome was investigated experimentally. Proteomic analysis was performed on secreted proteins harvested by trichloroacetic acid (TCA) precipitation, from the same cultures as outlined above for RNAseq and cytoplasmic proteomic analyses. Again, pairwise comparisons were generated for data analysis, i.e., 34°C versus 37°C and 37°C versus 40°C. Proteins displaying a minimum of a 2-fold change were identified and clustered by function. In contrast to the cytoplasmic proteome, the secreted proteome displayed large variations in response to changes in temperature. DEA between 34°C and 37°C revealed 427 proteins with a ≥2-fold change in abundance. Of these 427 proteins, 388 are increased in abundance at 34°C and 39 have increased abundance at 37°C (Fig. 4 and Data Set S3). DEA between 37°C and 40°C revealed 212 proteins with a ≥2-fold change in abundance. Of these 212 proteins, 207 show an increased abundance at 37°C and only 5 are increased in abundance at 40°C (Fig. 4 and Data Set S3). Similar to the trend observed in the RNAseq analysis, a large number of proteins display a decrease in abundance as temperature increases, while a relatively small number show increased abundance at increased temperatures. While a number of known secreted virulence factors display altered abundance in this proteomic analysis, many proteins found to be altered in the secreted fraction are typically considered cytoplasmic proteins. The presence of cytoplasmic proteins in such secreted proteomic analyses is a commonly observed phenomenon (21). Functional clustering revealed that in both comparisons, the majority of altered proteins are involved in energy metabolism. Proteins involved in nucleotide and protein synthesis are also among the most altered proteins and show increased abundances at lower temperatures in the secreted fraction.

10.1128/mSphere.01303-20.3 DATA SET S3 Secreted proteomics data analysis. Download Data Set S3, XLSX file, 0.2 MB. Copyright © 2021 Bastock et al. 2021 Bastock et al. https://creativecommons.org/licenses/by/4.0/ This content is distributed under the terms of the Creative Commons Attribution 4.0 International license.

### Posttranscriptional regulation of S. aureus proteins in response to temperature.

Parallel transcriptomic and proteomic analyses provided a valuable opportunity to examine the relationship between temperature-induced changes in protein and transcript levels in S. aureus. Specifically, it provided an opportunity to identify genes whose expression is subject to temperature-dependent posttranscriptional regulation. To perform this analysis, both proteomic data sets were combined (i.e., cytoplasmic and secreted), and all proteins demonstrating altered abundance (>3-fold) were identified. The transcriptomic data (corresponding to the same temperatures) were then examined to ascertain whether the variation in protein abundance could be accounted for by a similar alteration in transcript level (>3-fold). Proteins demonstrating temperature-dependent alterations in abundance for which the corresponding transcript does not mirror the observed change are deemed to be subject to posttranscriptional regulation. This analysis was performed twice, once for 34°C versus 37°C and again for 37°C versus 40°C. We employed a stricter fold-cutoff for this analysis (3-fold) to focus on genes/proteins demonstrating large changes in abundance based on the null hypothesis that proteins demonstrating large changes in abundance would always be accompanied by similar changes in transcript abundance.

**FIG 5:**
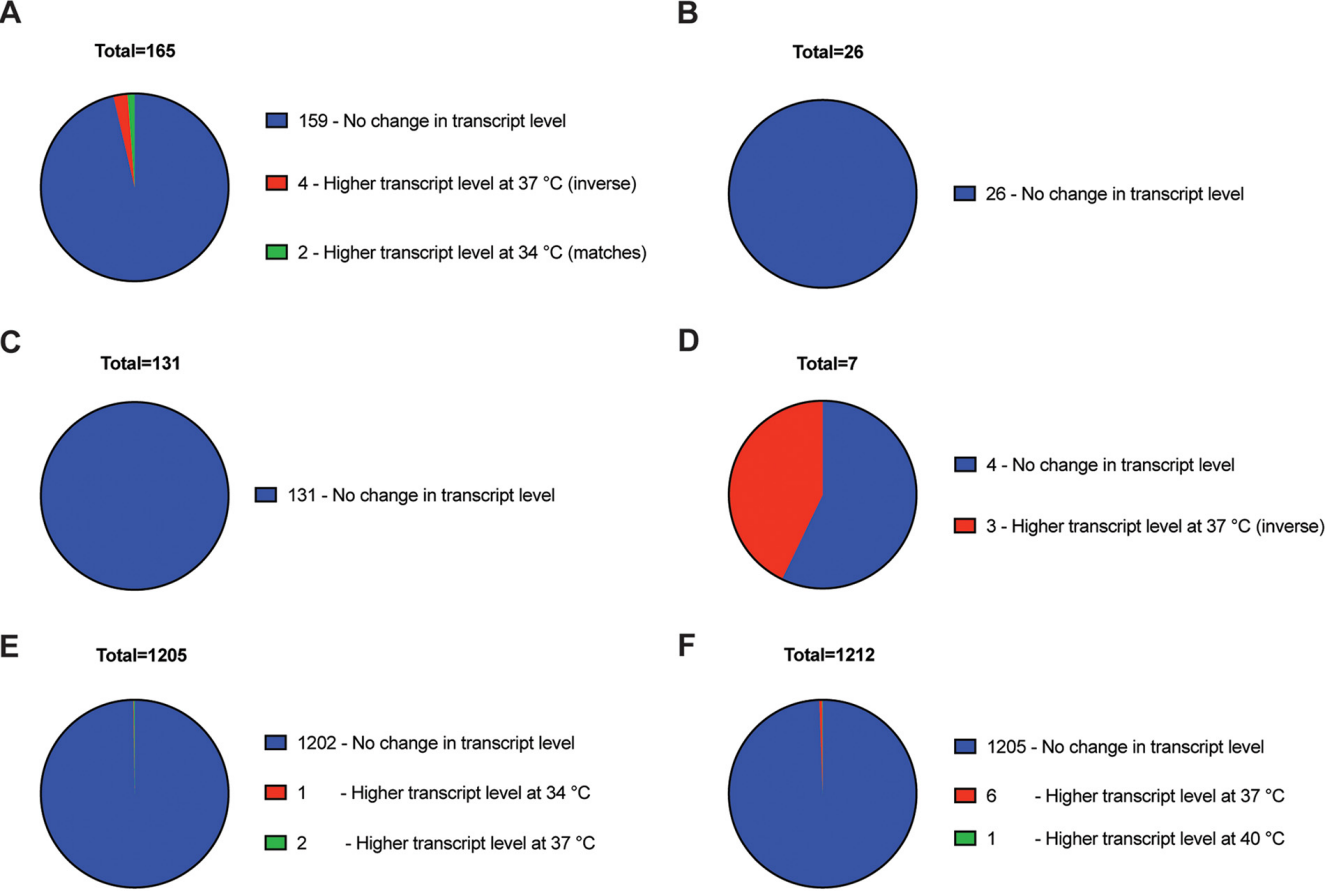
Correlation between temperature-dependent changes in protein and transcript levels in S. aureus. (A to D) Proteins that exhibit >3-fold changes in abundance at 34°C versus 37°C or 37°C versus 40°C were identified and their expression level determined using RNAseq data. Transcript level was then compared to protein level. (A) 165 proteins were found at higher levels at 34°C versus 37°C. 159 had no change in transcript level at the equivalent temperatures, 4 had higher transcript level at 37°C, and 2 have higher transcript level at 34°C (which correlates with protein levels). (B) 26 proteins were found at higher levels at 37°C versus 34°C. No changes in transcript level were observed for any of the 26 proteins. (C) 131 proteins were found at higher levels at 37°C versus 40°C. No changes in transcript level were observed for any of the 131 proteins. (D) 7 proteins were found at higher levels at 40°C versus 37°C. Four had no change in transcript level, while 3 had higher transcript levels at 37°C. (E and F) Proteins that did not exhibit changes in abundance at 34°C versus 37°C or 37°C versus 40°C were identified, and their expression level was determined using RNAseq data. Transcript level was then compared to protein level. (E) 1,205 proteins did not show differences in abundance between 34°C versus 37°C. 1,202 had no change in transcript level, 1 had higher transcript at 34°C, and 2 had higher transcript at 37°C. (F) 1,212 proteins did not show differences in abundance between 37°C versus 40°C. 1,205 of them had no change in transcript level, 6 had higher transcript at 37°C, and 1 had higher transcript at 40°C.

When comparing 34°C to 37°C, a total of 191 proteins were altered, with 165 found at higher levels at 34°C (Fig. 5A and B). For these 165 proteins, 159 of the corresponding genes showed no variation in transcript levels (or a variation <3-fold). Four genes showed an inverse relationship with transcript levels, while only two genes showed a variation in transcript level that mirrored that observed for the protein (Fig. 5A). Also in the comparison of 34°C to 37°C, 26 proteins were found in increased abundance at 37°C. For these 26 proteins, none demonstrated variation in transcript level greater than 3-fold (Fig. 5B). Collectively, these results show that for the 191 proteins with a >3-fold variation in abundance between 34°C versus 37°C, only 2 showed variation in transcript levels that could potentially explain the observed difference in protein abundance. These data suggest that expression of the genes encoding the remaining 189 of the 191 proteins is subject to temperature-responsive posttranscriptional regulation.

A similar comparison between 37°C versus 40°C revealed a total of 138 proteins altered, with 131 found increased in abundance at 37°C. For the 131 proteins with increased abundance at 37°C, none of the corresponding transcripts showed alterations (Fig. 5C). Also between 37°C versus 40°C, 7 proteins were found at an increased abundance at 40°C. For these 7 proteins, 4 corresponding transcripts showed no variation, while 3 (all part of the same operon) showed decreased abundance at 40°C (Fig. 5D and Data Set S4). Consequently, for none of the 138 proteins that were altered between 37°C and 40°C could this alteration be explained by variation in transcript level alone, suggesting that expression of these genes is subject to temperature-responsive posttranscriptional regulation.

10.1128/mSphere.01303-20.4 DATA SET S4 Transcriptomics/proteomics cross reference analysis. Download Data Set S4, XLSX file, 0.08 MB. Copyright © 2021 Bastock et al. 2021 Bastock et al. https://creativecommons.org/licenses/by/4.0/ This content is distributed under the terms of the Creative Commons Attribution 4.0 International license.

To further investigate temperature-responsive posttranscriptional regulation in S. aureus, it was determined whether similar amounts of protein can be produced from various amounts of transcript. To do so, proteins that did not show large variations in abundance (<3-fold) at 34°C versus 37°C and 37°C versus 40°C were identified and the transcript levels for the corresponding genes were examined. Results show that when protein levels were constant at different temperatures, transcript levels largely mirrored this pattern (Fig. 5E and F). For 34°C versus 37°C, 1,205 total proteins showed low/no variation in abundance. A total of 1,202 of the corresponding genes similarly demonstrated low/no variation in abundance (or variation <3-fold). Similar results were obtained for 37°C versus 40°C, with 1,205 genes corresponding to 1,212 proteins showing low/no variation in abundance.

Collectively, the results above show that (i) protein production in S. aureus is subject to a large degree of temperature-responsive posttranscriptional regulation, (ii) the amount of protein produced from identical/similar amounts of transcript is variable, and (iii) in general when protein level is relatively constant, transcript level of the corresponding gene is unchanged. This is the first demonstration of the extent and degree to which S. aureus genes are subject to temperature-responsive posttranscriptional regulation.

### αPSM production is influenced by temperature.

The repertoire of S. aureus secreted and cell wall-associated virulence factors includes a group of cytolytic peptides called alpha phenol-soluble modulins (αPSMs). In most S. aureus genome annotation files, the genes encoding the αPSM peptides are not annotated and therefore are often overlooked during transcriptomic analyses. Consequently, relatively little is known about the expression and regulation of the gene encoding the αPSMs. The analysis performed in this study utilized an updated genome annotation file, which includes the αPSM transcript annotation and thus generated expression data for the transcript. As outlined above, αPSM transcript levels were found to be influenced by temperature, with increased levels detected as temperature increases from 34°C to 37°C (2.38-fold) and from 37°C to 40°C (1.37-fold). The overall increase in αPSM transcript levels from 34°C to 40°C is 3.25-fold (Table 2 and Data Set S1). In the secreted proteome, αPSM1 and αPSM3 also increase from 34°C to 37°C (1.62-fold and 2.61-fold, respectively) and from 37°C to 40°C (1.20-fold and 1.74-fold, respectively), suggesting temperature-responsive regulation at the transcriptional level of αPSM production (Data Set S3). Despite being translated from the same transcript, αPSM4 showed no change in abundance between temperatures and αPSM2 was not identified in the secreted fraction at all (Data Set S3).

10.1128/mSphere.01303-20.1 DATA SET S1 RNAseq data analysis. Download Data Set S1, XLSX file, 0.03 MB. Copyright © 2021 Bastock et al. 2021 Bastock et al. https://creativecommons.org/licenses/by/4.0/ This content is distributed under the terms of the Creative Commons Attribution 4.0 International license.

**FIG 6:**
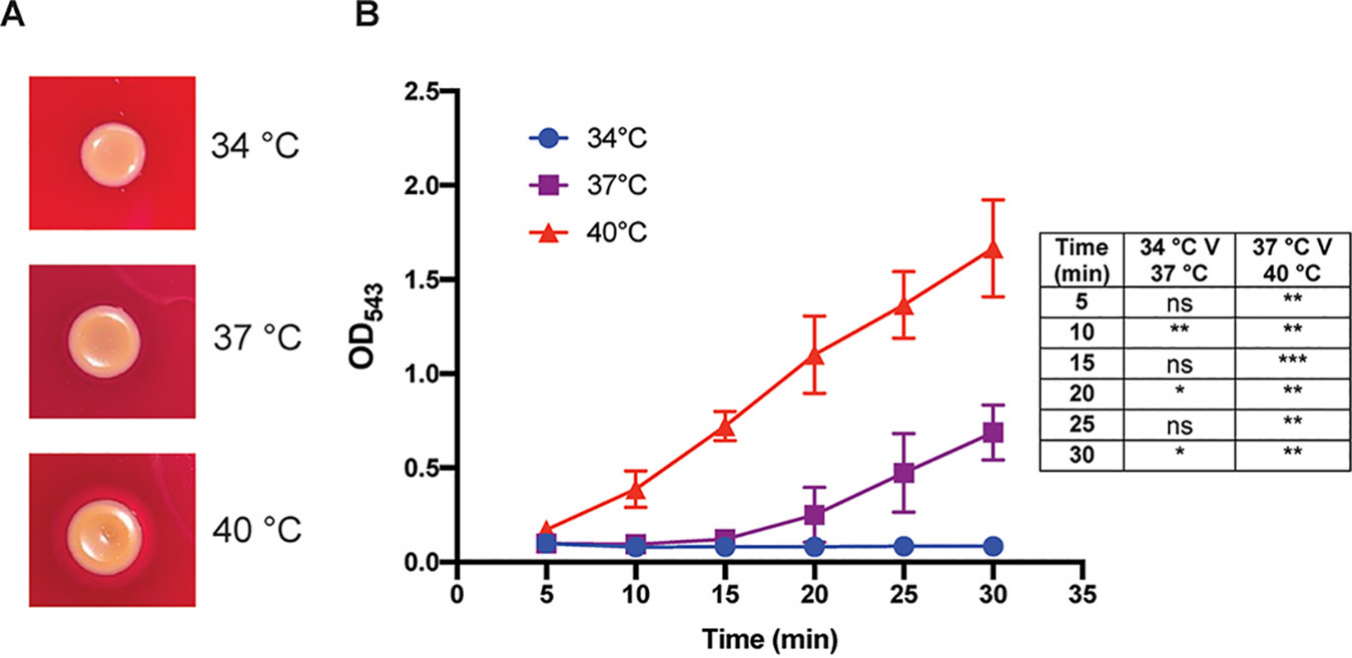
S. aureus hemolytic activity increases as temperature is increased. (A) 5 μl of overnight cultures were spot plated on 5% whole human blood agar plates and incubated at respective temperatures overnight. S. aureus incubated at 34°C (top) had little hemolytic activity compared to that incubated at 37°C (middle) and 40°C (bottom). S. aureus incubated at 40°C had the most hemolytic activity. (B) Cell-free culture supernatants of S. aureus were incubated with whole human blood for the indicated times. Supernatants from cultures at 40°C show a significant increase in hemolytic activity over the entire 25 min incubation period. Supernatants from cultures at 34°C have significantly less hemolytic activity relative to those at 37°C at 10, 20, and 30 min. *, *P* value < 0.05; **, *P* value < 0.01; ***, *P* value < 0.001; ns, not significant. Statistical analyses were performed using a Student’s *t* test.

To confirm that the transcriptomic and proteomic data manifest as a biologically meaningful phenotype, αPSM production/activity at 34°C, 37°C, and 40°C was experimentally examined. Wild-type S. aureus was spotted on 5% whole human blood agar plates and incubated overnight at the respective temperatures. Hemolysis of human erythrocytes by S. aureus is primarily attributed to the αPSMs (22); thus, a zone of clearing around the colony is suggestive of αPSM activity and abundance. A clear temperature-responsive trend was observed with little to no zone of hemolysis visible around the colony incubated at 34°C, while a large zone of clearance was observed around the colony incubated at 40°C (Fig. 6A). Incubation at 37°C resulted in an intermediate phenotype. These results demonstrate that a clear difference in hemolytic activity exists at the three different temperatures. These data support the finding of increased αPSM levels at increased temperatures.

The result above suggests that αPSM production is dependent on temperature; however, to account for potential differences in the observed phenotype due to variation in growth at the three temperatures, we performed a whole human blood lysis assay (23) to quantify the observed temperature-dependent differences in hemolytic activity. S. aureus was grown for 17 h at the three temperatures and cell-free supernatants were incubated with whole human blood (as outlined above, S. aureus cultures at each temperature reached the same OD_600_ in stationary phase). Over a course of 30 min, the degree of hemolysis was assessed every 5 min. Hemolytic activity of supernatants from cultures grown at 40°C is significantly increased at all time points compared to that of supernatants from cultures grown at 37°C (Fig. 6B). At most time points tested, supernatants from cultures grown at 37°C show significantly higher hemolytic activity than those from cultures grown at 34°C. Collectively, these data validate the transcriptomic and proteomic data sets and demonstrate that the temperature-responsive alterations in αPSM transcript and protein levels observed manifest as biologically meaningful alterations in toxin activity. To our knowledge, this is the first demonstration that alterations in temperature influence αPSM production in S. aureus.

### Production of staphyloxanthin is increased at higher temperatures.

**FIG 7:**
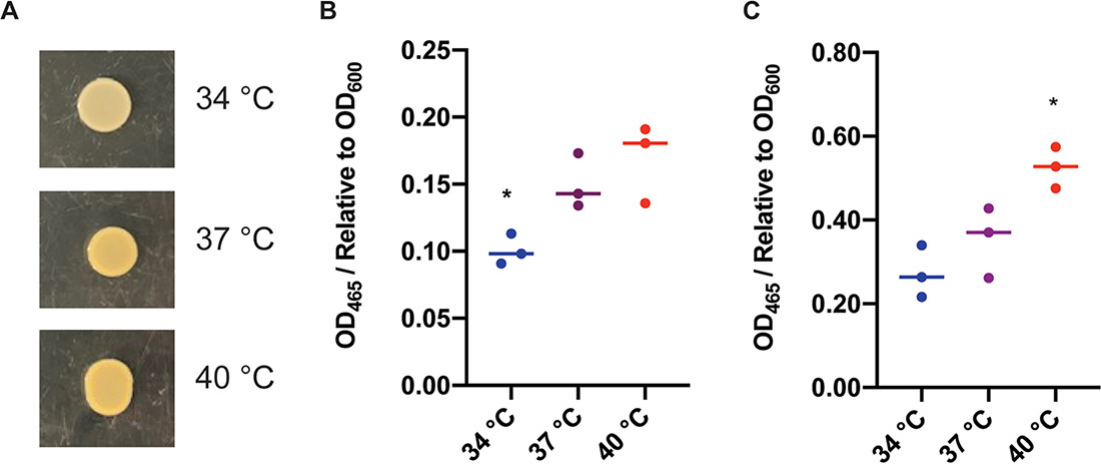
S. aureus pigmentation is increased as temperature increases. (A) Wild-type (WT) S. aureus was spot plated on TSA and incubated at 34°C, 37°C, and 40°C overnight. (B) Staphyloxanthin quantification from colonies grown on TSA plates. Wild-type S. aureus was spot plated on TSA and incubated at the indicated temperature overnight. Colonies were resuspended in water and the OD_600_ was read. Methanol was then used to extract the staphyloxanthin from samples and the OD_465_ was read. Samples were normalized to the readings from the 37°C samples. Pigmentation is significantly decreased at 34°C, and a nonsignificant increase was observed at 40°C. (C) Staphyloxanthin quantification from liquid cultures. WT S. aureus was incubated overnight in TSB at the respective temperatures, and quantification of staphyloxanthin was performed as described above. In liquid culture, pigmentation is significantly higher at 40°C. *, *P* value < 0.05. Statistical analyses were performed using a Student’s *t* test.

In addition to a difference in hemolytic activity, colonies of wild-type S. aureus incubated overnight on human blood agar plates displayed a difference in pigmentation at the three respective temperatures (Fig. 6A). This phenotype was confirmed on tryptic soy agar (TSA) agar plates (Fig. 7A). Similar to the temperature-responsive trend on the human blood agar plates, colonies showed an increase in pigmentation as temperature increased. To further investigate and quantify pigment production at different temperatures, a quantitative pigmentation assay was performed as described by Austin et al. (24). This assay was then modified to quantify pigmentation in a liquid-based culture. As observed in Fig. 6A and Fig. 7A, results show a temperature-dependent increase in pigmentation from 34°C to 37°C and a further increase from 37°C to 40°C in both colony- and liquid-based analyses (Fig. 7B and C).

Pigmentation in S. aureus is due to the molecule staphyloxanthin, which has previously been shown to be a major contributor to virulence. To investigate whether staphyloxanthin production is temperature regulated, the RNAseq data were first examined to determine expression levels of the *crtOPQNM* operon at different temperatures. Interestingly, no notable difference in *crtOPQNM* expression was observed. There was also no difference observed in the levels of two corresponding proteins, CrtP (34°C versus 37°C fold change 0.959, 37°C versus 40°C fold change 0.849) and CrtM (34°C versus 37°C fold change 0.614, 37°C versus 40°C fold change 1.289), in the cytoplasmic proteomic analysis (Data Set S2). Interestingly, the other corresponding proteins (CrtO, CrtQ, and CrtN) were not observed in the cytoplasmic proteomic analysis. Since no differences in *crtOPQNM* expression or the corresponding proteins were observed, it is possible that the activity of the enzymes in the staphyloxanthin biosynthetic pathway could be sensitive to temperature.

### Aureolysin activity is increased at lower temperatures.

**FIG 8:**
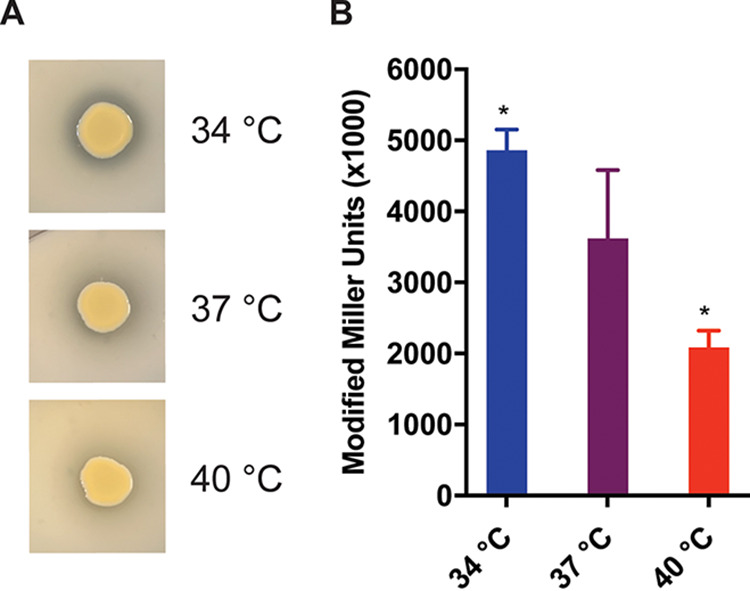
Aureolysin production is decreased as temperature increases. (A) WT S. aureus cultures were spot plated on 15% casein TSA plates and incubated at the respective temperatures for 48 h. A larger zone of clearing is suggestive of greater aureolysin abundance. Incubation at 34°C had a large zone of clearing, while incubation at 40°C had relatively none. (B) RKC0654 cultures were grown at the indicated temperature until mid-log phase, when aureolysin is known to be highly expressed. Supernatants were collected and filter sterilized for β-galactosidase assays. Cultures grown at 34°C displayed a significant increase in β-galactosidase activity compared to those grown at 37°C and 40°C, indicating increased activity of the aureolysin promoter at this temperature. *, *P* value < 0.05; **, *P* value < 0.01; ***, *P* value < 0.001. Statistical analyses were performed using a Student’s *t* test.

The metalloprotease aureolysin plays a critical role during S. aureus infection. The functionality of this protease has been widely studied; however, little is known about its regulation. Data from the RNAseq analysis, as well as the secreted proteome, indicate that aureolysin is subject to temperature-dependent regulation in an inverse fashion (i.e., decreased production as temperature increases). To further investigate and quantify the impact of temperature on aureolysin production/activity, S. aureus was inoculated on casein agar plates and incubated overnight at 34°C, 37°C, and 40°C. Colonies on plates incubated at 34°C had a large zone of clearing, indicating high levels of caseinase activity by aureolysin. The zone of clearing was decreased on plates incubated at 37°C and was barely detectable at 40°C (Fig. 8A). These results confirm the proteomic and transcriptomic data and suggest that aureolysin protein levels, and subsequently activity, are inversely correlated with temperature.

As indicated above, both transcriptomic and proteomic data analyses demonstrate temperature-dependent alterations in *aur* mRNA and aureolysin levels, respectively, with decreased levels of both observed at increased temperatures. To further characterize this regulation, an *aur-lacZ* reporter construct was used to quantify aureolysin expression at different temperatures. This construct places *lacZ* expression under the regulation of the *aur* native promoter and 5′ untranslated region (5′ UTR). Cultures were incubated to late-log phase (when protease production is induced) and LacZ was quantified by β-galactosidase assay as previously described (24). Results show that *aur-lacZ* expression is temperature-dependent in a manner identical to that observed in the transcriptomic/proteomic data and casein agar plate assay, i.e., higher levels of promoter activity at 34°C and decreasing activity at 37°C and 40°C (Fig. 8B). Collectively, the data indicate that *aur* is subject to temperature-dependent regulation mediated by sequences within the promoter and 5′ UTR of the gene and are consistent with the alterations observed in the omics analyses. Increased aureolysin production and activity at lower temperatures may contribute to colonization or asymptomatic carriage in the nares.

### S. aureus demonstrates increased invasion of epithelial cells at 37°C.

Results above demonstrate that (i) S. aureus gene expression is affected by slight changes in temperature and that (ii) temperature-dependent alterations in gene expression manifest as biologically meaningful alterations in the activity of virulence factors. These temperature-responsive variations in virulence factor activity would be predicted to affect S. aureus colonization and pathogenesis *in vivo*. To test this hypothesis, RPMI 2650 cells (a human nasal epithelial cell line [HNEC]) were used to measure the ability of S. aureus to invade and survive intracellularly at both 34°C and 37°C. RPMI 2650 cells were grown at 34°C or 37°C prior to infection with wild-type S. aureus also cultured at 34°C or 37°C. The RPMI 2650 cells were subsequently infected with the culture of the same temperature at a multiplicity of infection (MOI) of 10 and incubated at that conserved temperature. At 0, 1, 6, and 24 h postinfection, RPMI 2650 viability was assessed using an MTT [3-(4,5-dimethyl-2-thiazolyl)-2,5-diphenyl-2H-tetrazolium bromide] viability assay, and intracellular bacterial load was determined by diluting and plating. This approach makes it possible to determine and compare both invasion of epithelial cells (*t* = 1 h) and survival of S. aureus in the intracellular environment (*t* = 6 h and *t* = 24 h) at 34°C and 37°C.

**FIG 9:**
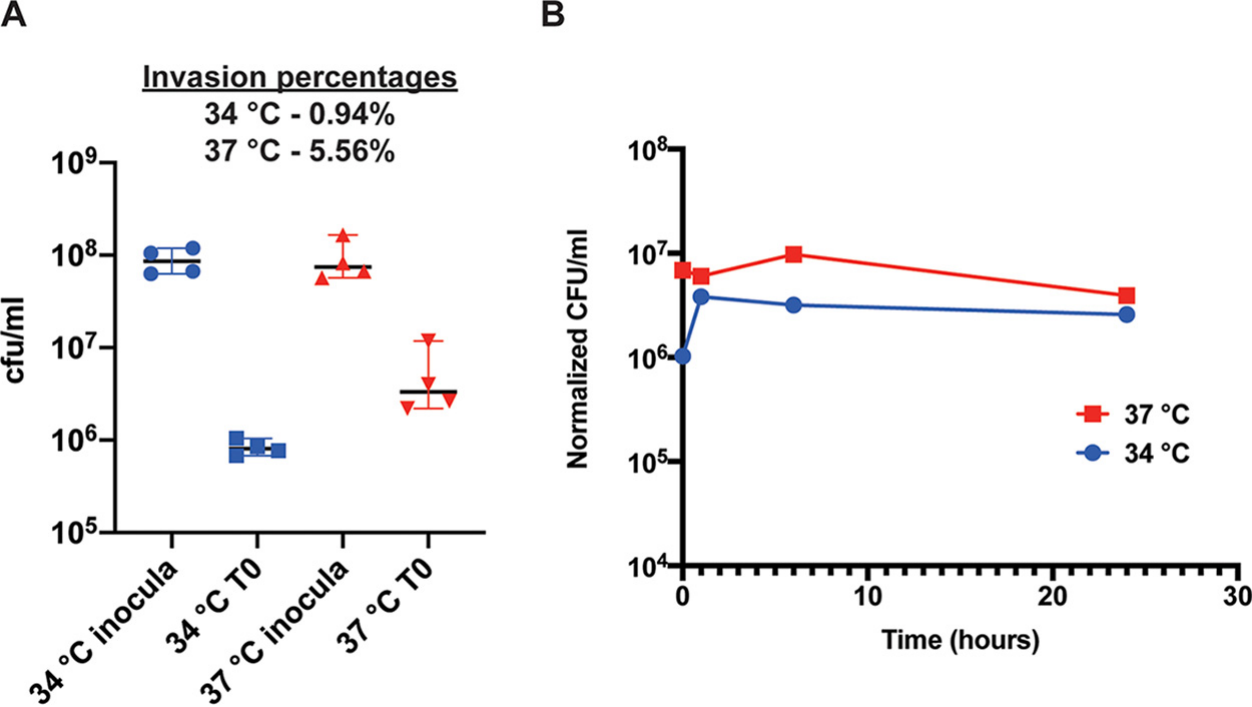
RPMI 2650 human nasal epithelial cell infection. HNECs were grown at 34°C and 37°C and infected with WT S. aureus grown at 34°C and 37°C. Infected cells were incubated at the corresponding temperature of 34°C or 37°C for the duration of the experiment. (A) Decreased intracellular invasion of S. aureus at 34°C. The S. aureus CFU/ml in each inocula was determined by serial dilution and plating and is shown for each inocula (34°C inocula and 37°C inocula). The number of bacterial cells that successfully entered the HNEC cells at time point zero is also indicated for each infection (34°C T0 and 37°C T0). At 34°C, 0.94% of the inocula invaded while at 37°C, 5.56% invaded. Invasion experiments were performed in technical quadruplicate on three separate occasions and representative data sets are shown. (B) Intracellular survival of S. aureus over time in HNECs. Bacterial load (determined by dilution plating samples at each temperature and time point) was normalized against the number of viable HNECs at each time point (determined by MTT assay), and the resulting values were plotted over time. Relative survival of bacteria inside HNEC cells was similar at 34°C and 37°C. Invasion experiments were performed on three separate occasions and representative data sets are shown.

Results demonstrate that invasion of RPMI 2650 cells by S. aureus is higher at 37°C than at 34°C. Roughly 5.5% of the initial inoculum was found within the intracellular environment at 37°C following a 1 h incubation. In contrast, <1% of the inoculum had invaded the RPMI 2650 cells at 34°C at the same time point (Fig. 9A). To examine intracellular survival/persistence of S. aureus, the bacterial load at each time point (determined by dilution plating) was normalized against the number of viable RPMI 2650 cells at each time point (determined by MTT assay). This approach controls for RPMI 2650 cell death at later time points. Once inside RPMI 2650 cells, survival of S. aureus appears to be comparable at both temperatures (Fig. 9B). These data suggest that S. aureus is less invasive toward RPMI 2650 cells at 34°C, the temperature associated with colonization, but is able to survive in the intracellular environment equally at both temperatures.

## DISCUSSION

S. aureus experiences small but defined changes in environmental temperature during the transition between colonization in the nares and infection within deeper tissues of the human body (19). To our knowledge, this study is the first to investigate global differences of gene expression in S. aureus cultured at 3 physiologically relevant temperatures and to investigate if these differences manifest as biologically meaningful changes in the bacteria’s pathology. The results overwhelmingly support the hypothesis that small temperature differences (e.g., a change in temperature from 34°C to 37°C) lead to relevant alterations of the S. aureus transcriptome and proteome. Phenotypic assays associated with altered levels of selected proteins validate that these changes do, in fact, have physiological significance. This study is also the first to show an altered invasive phenotype of S. aureus at 34°C versus 37°C (nares and body temperatures, respectively) using a cell culture infection model.

Although substantial differences were observed in the transcriptome and proteome of S. aureus at the three temperatures tested, the differences were especially notable in comparisons of 34°C versus 37°C, the environmental temperatures associated with colonization and infection, respectively (Fig. 2 and 4 and see Data Sets S1 and S3 in the supplemental material). Interestingly, the majority of transcripts and proteins that were altered in this comparison exhibited increased expression at the lower temperature, including the proteases SspA, SspB, and aureolysin. One proposed function of these proteases is to prevent opsonization and phagocytosis of the bacterium by inhibiting various components of the innate immune system (25–30). Based on this, we anticipated that protease abundance would be increased at 37°C (invasive infection) compared to that at 34°C (colonization). It was therefore surprising to find increased levels of SspA, SspB, and aureolysin at 34°C. Work by Kolar et al. demonstrated that S. aureus proteases mediate virulence by regulating the amount of functional secreted virulence factors (25). Consequently, increased protease production at 34°C could serve to (i) protect the bacteria from immune components present in the mucous membranes (such as antimicrobial peptides) and (ii) dampen virulence by degrading secreted virulence factors, therefore promoting a colonization phenotype.

Previous work by our group characterized an unconventional RNA thermosensor in the 5′ UTR of the *cidA* transcript (31). This thermosensor mediates posttranscriptional temperature-dependent gene regulation that leads to increased translation at lower temperatures. This pattern of regulation (i.e., increased translation at 34°C compared to that at 37°C) is remarkably similar to the overall pattern of regulation observed in this study (i.e., increased protein abundance at lower temperatures). The proteomic/transcriptomic cross reference analysis outlined in Fig. 5 illustrates that a large number of S. aureus genes undergo temperature-dependent posttranscriptional regulation. It is tempting to speculate that some of these genes may be regulated by thermosensors similar to that found in the *cidA* 5′ UTR and that this (or alternative) mechanism(s) of posttranscriptional temperature-dependent gene regulation may be widespread in S. aureus. Studies are ongoing to determine the contribution of such thermosensors in mediating the temperature-dependent posttranscriptional regulation identified in this global study.

Although a variety of S. aureus genes showed temperature-dependent variation in expression, functional clustering of the RNAseq data revealed that sRNAs were consistently the most variable (29 sRNA genes altered at 34°C versus 37°C; 18 altered at 37°C versus 40°C), with 15 sRNAs displaying a 2-fold change in expression between both DEAs (Fig. 2, Table 1, and Data Set S1). These data suggest that sRNAs represent key regulatory molecules that allow S. aureus to rapidly adapt as it transitions between the nares and the body. Although the regulatory roles of most of the sRNAs identified in this study are unknown, some have been shown to play a role in virulence via *trans-*acting posttranscriptional regulatory mechanisms. For example, sprD was previously shown to repress the translation of Sbi, an immune evasion protein (32, 33). The data in this study support this result, as sprD and *sbi* transcript levels were found to be increased at 34°C, while Sbi protein levels were decreased at this temperature. These data suggest that at 34°C, sprD is repressing Sbi translation and that this repression is relieved at higher temperatures (when sprD levels decrease). Interestingly, a study by Bordeau et al. showed increased production of sprD in colonization isolates compared to that in sepsis isolates (34). We hypothesize that sprD may be contributing to colonization of S. aureus by repressing Sbi production, which, in turn, helps maintain a commensal relationship with the host. Teg49 is another sRNA that was identified as being temperature regulated in this study that has been shown to play a role in S. aureus virulence (35, 36). Located in the promoter region of the gene encoding staphylococcal accessory regulator A (SarA), Teg49 has been shown to regulate virulence factor gene expression independent of SarA activity (35, 36). Interestingly, both SarA transcript (1.61-fold change between 34°C versus 37°C and 1.71-fold change between 37°C versus 40°C) and protein (1.12-fold change between 34°C versus 37°C and 0.79-fold change between 37°C versus 40°C) levels remain relatively constant between the temperatures despite the ∼10-fold change in Teg49 expression between each DEA. It is plausible that other temperature-responsive sRNAs in this list are responsible for the observed differences in the proteome, thus facilitating temperature adaptation by S. aureus.

The primary objective of this study was to determine whether small variations in environmental temperature affect gene expression in S. aureus, and the results generated clearly demonstrate that this is the case. To determine whether the observed temperature-dependent changes in gene expression manifest as biologically meaningful differences in S. aureus pathogenesis, a nasal epithelial cell infection model was employed. Intracellular invasion and persistence of S. aureus has been characterized in various cell types (37–44), but to our knowledge the influence of temperature on colonization and invasion has not been previously investigated. We elected to perform these studies in a human nasal epithelial cell line, representative of the environment found in the human nares. Although intracellular persistence of S. aureus at 34°C and 37°C was comparable, the data indicate reduced intracellular invasion of S. aureus at 34°C compared to that at 37°C. These data clearly demonstrate that environmental temperature affects S. aureus pathogenesis in an *in vivo* assay. While the molecular factors responsible for the decreased invasion at 34°C are not clear, we hypothesize that the reduced invasion of S. aureus cells at 34°C aids in maintenance of an extracellular lifestyle during colonization.

Collectively, our results demonstrate that small, physiologically relevant changes in environmental temperature influence S. aureus gene expression, which we hypothesize influences the transition between the commensal and invasive states of the pathogen. We acknowledge a number of caveats that should be taken into consideration regarding this study, including the fact that transcript and protein stability was not assessed. It is possible that some of the differences observed result from transcript or protein instability at higher temperatures. While possible, we expect stability differences to be minimal, as the temperature range being studied was relatively small (i.e., a total of 6°C from 34°C to 40°C) and global temperature-dependent changes were not observed. Consequently, we expect the number of transcripts/proteins that may actually be affected by differential stability to be small. Another consideration of the secreted proteome is the identification of predicted cytoplasmic proteins in the secreted fraction. While this is a somewhat common phenomenon for the secretome of S. aureus (21), a factor that was not addressed in this study was membrane fluidity. Membrane fluidity is known to be affected by environmental temperature, but the degree of its effect on S. aureus was not addressed in this study. Typically, membrane fluidity increases at higher temperatures, which may give rise to increased secretion/leakage of protein from the cytoplasm. However, the general trend observed in this study was increased abundance of proteins at lower temperatures; therefore, we feel it is unlikely that these differences could be accounted for by variations in membrane fluidity. The release of proteins from the bacterial cell by extracellular vesicles (EVs) has recently been demonstrated in S. aureus, and the αPSM peptides are known to positively influence EV production (45). Since αPSM production was higher at higher temperatures (Fig. 6), we hypothesize that EV production may also be increased at higher temperatures. Nonetheless, this is unlikely to have influenced the observations in this study of increased protein secretion at lower temperatures.

In conclusion, this study clearly demonstrates that temperature is an underappreciated factor that influences gene expression and pathogenesis in S. aureus. To our knowledge, it is the first demonstration that temperature changes can have a meaningful physiological effect both *in vitro* and *in vivo.* These results have important implications in a variety of research areas, including the transition of S. aureus from commensal to pathogen and the interactions of S. aureus with other members of the nasal microbiome. Consideration should be given to the temperature at which these types of studies are performed.

## MATERIALS AND METHODS

### Strains, strain construction, and plasmids.

All strains and plasmids used in this study are listed in Table 3. To construct strain RCK0654, plasmid pCK3 (24) was electroporated into RN4220 (46), reisolated, and electroporated into USA300 AH1263 (47). A similar process was used to introduce the promoterless *lacZ* fusion plasmid pJB185 (48) into USA300 AH1263 to generate RKC0660.

**TABLE 3:** Strains and plasmids

Strain or plasmid	Characteristics	Source or reference
Strains		
S. aureus		
AH1263	USA300 LAC isolate cured of plasmid LAC-p03	(47)
RN4220	Restriction-deficient transformation recipient	(46)
RKC0654	AH1263 pCK3	This study
RKC0660	AH1263 pJB185	This study
E. coli		
DH5α	Cloning strain	Invitrogen
Plasmids		
pCK3	P*_aur_-lacZ* reporter plasmid; Amp^r^ Cm^r^	(24)
pJB138	Promoterless codon-optimized *lacZ* reporter plasmid; Amp^r^ Cm^r^	(24)

### Bacterial growth conditions.

S. aureus cultures were grown with shaking at 34°C, 37°C, or 40°C in tryptic soy broth (TSB). When appropriate, the antibiotic chloramphenicol was added to the media for a concentration of 10 μg ml^−1^. Escherichia coli DH5α cultures were grown at 37°C with shaking in lysogeny broth (LB). When appropriate, the antibiotic ampicillin was added to the media for a concentration of 100 μg ml^−1^. When synchronization of cultures was necessary for comparative analysis, 5 ml of overnight starter cultures was diluted 1:100 into 10 ml of fresh TSB. The diluted cultures were grown for 3 h until midexponential phase and then were subsequently diluted in 25 ml of fresh, prewarmed TSB to obtain an optical density at 600 nm (OD_600_) of 0.05. Cultures were incubated at the respective temperatures for the time indicated with each experiment.

### Preparation of RNA and protein samples for transcriptomics/proteomics.

S. aureus was grown in triplicate, in 5 ml overnight cultures with shaking at 37°C. The next day, cultures were diluted 1:100 in 25 ml of TSB and grown with shaking at 34°C, 37°C, or 40°C. Cultures at 34°C were inoculated 1 h prior to those at 37°C and 40°C to account for the slower growth at this temperature. Cultures were grown to postexponential phase (6 h for cultures at 37°C and 40°C, 7 h for cultures at 34°C), and 4 ml was collected, washed with ice-cold phosphate-buffered saline (PBS), and stored at −80°C for RNA isolation. Eighteen ml of the remaining cell culture was collected and centrifuged at 3,000 rpm for 15 min. Supernatants were harvested and filter sterilized through a 0.45 μm filter disk to ensure that all bacterial cells were removed from the sample. The remaining cell pellets were washed with ice-cold PBS and stored at −80°C for intracellular proteome analysis. Trichloroacetic acid (TCA) was added to the culture supernatants (for a final concentration of 10%) and samples were incubated at 4°C overnight. The following day, samples were centrifuged at 11,000 rpm for 10 min and the supernatant was removed. The resulting pellets, containing precipitated proteins, were washed with ice-cold acetone and sent to the University of Nebraska Lincoln Proteomics Facility for mass spectrometry analysis.

To prepare samples for intracellular proteome analysis, pelleted bacteria were washed, resuspended in 1 ml phosphate-buffered saline (PBS) containing 30 μl of lysostaphin, and incubated statically at 37°C for 15 min. Samples were removed and homogenized for 30 s, followed by another 15 min incubation period at 37°C. This process was repeated 4 times. Samples were spun by centrifugation to pellet cell debris. The lysate was removed and sent to the University of Nebraska Lincoln Proteomics Facility for mass spectrometry analysis.

### Mass spectrometry proteomics and data analysis.

Cytoplasmic protein supernatants (100 μl) were diluted to 200 μl with water and precipitated and washed 3 times using the ProteoExtract Protein Precipitation kit (EMD Millipore). Both secreted and supernatant pellets were redissolved in a solution of 7 M urea, 2 M thiourea, 5 mM dithiothreitol (DTT), 0.1 M Tris (pH 8), and 1× PhosStop by gentle shaking for 1.5 h at 24°C. After reduction, the samples were alkylated for 30 min using a 3-fold molar excess of iodoacetamide. The protein concentration was determined using the CB-X protein assay kit. One hundred μg of protein in 10 μl of the urea solution was diluted and digested with 1 μg trypsin (1:100 enzyme to substrate ratio) for 16 h overnight, and then an additional 1 μg of trypsin was added and digestion was continued for a further 3 h. Five hundred ng of each of the 9 secreted protein samples and 1 μg of each of the cytoplasmic protein samples was run by nano LC-MS/MS using a 2 h gradient on a 0.075 mm by 250 mm C_18_ Waters CSH column feeding into a Q-Exactive HF mass spectrometer as described previously (49). All MS/MS samples were processed using Proteome Discoverer 2.2 software (Thermo) linked to the Mascot Server MS/MS search software (Matrix Science, London, UK, version 2.6.1). Mascot was set up to search the common contaminants database cRAP_20150130.fasta (117 entries) and the uniprot-S_aureus database (2,607 entries). Mascot was searched with a fragment ion mass tolerance of 0.06 Da and a parent ion tolerance of 10.0 ppm. Oxidation of methionine, acetylation of N-term protein, deamination of asparagine and glutamine, phosphorylation of serine, threonine, and tyrosine, and carbamidomethylation of cysteine were specified as variable modifications. The data were searched using a decoy database to set the false discovery rate to 1% (high confidence). The protein quantification was processed as described previously (49). The mass spectrometry proteomics data have been deposited to the ProteomeXchange Consortium via the PRIDE (50) partner repository with the data set identifier PXD023039.

### RNA isolation and rRNA removal.

RNA was isolated as described previously by our lab (51) from the 4 ml pellets indicated above. rRNA (rRNA) was removed using a combination of the MICROBExpress kit (Invitrogen) and a novel protocol developed in this study, called riboDON (depletion of nucleic acid). The riboDON protocol was specifically designed to remove excess 23S rRNA from samples as follows. A PCR product corresponding to the S. aureus 23S rRNA gene was generated using forward primer 791 and reverse primer 791 containing biotin at the 5′ end. The resulting DNA product has one biotinylated strand corresponding to the sequence antisense to the 23S rRNA. Magnetic beads (Dynabeads M-280, Invitrogen) were washed according to the manufacturer’s instructions and resuspended in 400 μl of 2× binding and washing (B&W) buffer (10 mM Tris-HCl [pH 7.5], 1 mM EDTA, 2 M NaCl). Biotinylated 23S PCR product (25 μg) in 400 μl water was added to the beads to bring the B&W buffer to 1×. Tubes were rolled gently overnight at room temperature to facilitate binding of the oligonucleotides to the beads. Tubes were then placed on a magnetic rack for 2 min and the supernatant was removed. Beads were washed 3× with 1× B&W buffer. The sense DNA strand was melted off by adding 100 μl of 100 mM NaOH to the beads and incubating for 10 min. Beads were washed once with 100 μl of 100 mM NaOH and then twice with 1 ml of 1× B&W buffer. Finally, beads were resuspended in 200 μl of 1× B&W buffer and kept at 4°C until use.

To remove rRNA from total RNA samples, RNA (4 μg) was initially treated with the MICROBExpress kit according to the manufacturer’s instructions. After precipitation and washing, RNA pellets were resuspended in 10 μl of water and 40 μl of binding buffer from the MICROBExpress kit. The riboDON beads (prepared as above) were captured on a magnetic rack, the supernatant was removed, and the 50 μl of MICROBExpress-treated RNA in binding buffer was added. The RNA and riboDON bead mixture was incubated at 37°C for 3 h in a rotating hybridization oven. Beads were captured, samples (containing rRNA depleted RNA) were removed, and RNA was precipitated and washed again. The final RNA pellet was resuspended in 3 μl of water, and 1 μl was analyzed on a Bioanalyzer RNA 6000 Nano chip (Agilent) to confirm rRNA removal.

### RNAseq.

RNAseq libraries were created by the Ohio University Genomics Facility using rRNA depleted RNA samples and the Illumina Truseq stranded mRNA library prep kit. Libraries were sequenced by the Nationwide Children’s Hospital Genomics Services Laboratory on an Illumina HiSeq 3000/4000 system using the TG HiSeq 3000/4000 PE ClusterKit for cluster generation and HiSeq 3000/4000 SBS kit (300 cycles) for sequencing. Data were exported and analyzed using CLC Genomics Workbench as described previously (51). Briefly, reads corresponding to rRNA were removed prior to analysis, and expression values were calculated for each gene using the RNAseq function. Differential expression analyses were performed comparing gene expression at different temperatures. Results were normalized using quantile normalization. Lowly expressed genes (i.e., where expression values were <10 in both conditions) were excluded, as were genes in which <80% of the mapped reads were nonunique. RNAseq data are deposited in the Gene Expression Omnibus (GEO) database under the accession number GSE162697.

### Cell-free human blood hemolysis assay.

Hemolytic activity of S. aureus culture supernatants against human erythrocytes was determined as described previously (23, 52). Synchronized, filter-sterilized S. aureus culture supernatants were diluted 1:2 in reaction buffer (40 mM CaCl_2_, 1.7% NaCl) and incubated at 37°C in a tube revolver with 25 μl of whole human blood. Following a 10 min incubation, the samples were centrifuged at 5,500 × *g* for 5 min, and 100 μl of the supernatant was transferred to a 96-well plate. The degree of erythrocyte lysis was determined by reading the absorbance of the samples at OD_543_. To measure hemolytic activity over time, six replicate reactions were set up for each sample. One replicate was taken at each of the indicated time points and centrifuged (5,500 × *g*, 5 min), and absorbance of supernatants was measured at OD_543_.

### Human blood agar plates.

Replicate TSA plates, containing 5% (vol/vol) whole human blood, were spot plated with 5 μl of S. aureus overnight cultures. Plates were then incubated overnight at 34°C, 37°C, or 40°C.

### Staphyloxanthin assay.

Quantification of pigment production at each temperature was performed using the method outlined by Austin et al. (24). S. aureus 5 ml cultures were grown in triplicate with shaking overnight at 37°C. The next day, cultures were diluted 1:100 into 25 ml of fresh, prewarmed tryptic soy broth (TSB) and grown overnight at 37°C. One ml of overnight cultures was pelleted by centrifugation at 21,130 × *g* for 10 min and resuspended in 1 ml H_2_O. The OD_600_ value of each sample was determined, and then bacterial cells were pelleted by centrifugation at 21,130 × *g* for 3 min. The supernatant was discarded, and the pellet was resuspended in 420 μl of methanol. The sample was vortexed and then incubated at 55°C for 5 min. After the indicated time, samples were centrifuged at 21,130 × *g* for 2 min, 100 μl of the supernatant was removed, and absorbance at 465 nm was measured. Pigmentation was quantified by dividing the OD_465_ by the relative OD_600._ Relative OD_600_ was obtained by dividing individual OD_600_ values by the average OD_600_ of cultures grown at 37°C, since this is the standard lab condition. To measure pigment production in colonies of S. aureus grown on agar plates, 25 μl of the synchronized, overnight cultures was spot plated onto replicate TSA plates and incubated at 34°C, 37°C, or 40°C. Colonies from the TSA plates were suspended in 1 ml H_2_O and the OD_600_ value of each sample was determined. Cells were then pelleted, resuspended in methanol, and processed as outlined above.

### Caseinase activity.

Casein agar plates (TSB agar containing 15% nonfat milk) were used as described previously (53). Casein plates were inoculated with 5 μl of S. aureus overnight cultures. Zones of clearing, resulting from casein digestion, were observed and photographed after 48 h of incubation at 34°C, 37°C, or 40°C.

### β-galactosidase assay.

β-galactosidase assays were performed as described previously (24). Cultures were grown in triplicate with shaking overnight at 37°C. The following day, cultures were diluted 1:100 into 25 ml of fresh, prewarmed TSB. Cultures grown at 34°C were inoculated 1 h before cultures grown at 37°C and 40°C to account for the lower growth rate. After 7 h of growth (time from inoculation of the 34°C cultures), 1 ml was withdrawn, centrifuged at 21,130 × *g* for 1 min, and then resuspended in 1.2 ml of Z-buffer. The cell suspension was homogenized using 0.1-mm-diameter glass beads. Cellular debris was pelleted by centrifugation at 21,130 × *g* for 5 min, and the lysate was transferred to a fresh 1.5-ml tube. An aliquot of lysate was removed and combined with Z-buffer for a total volume of 700 μl. To this, 140 μl of reaction mix was added (*o*-nitrophenyl β-d-galactopyranoside [ONPG] [4 mg ml^−1^], 40 mM NaH_2_PO_4_, and 60 mM Na_2_HPO_4_ [pH 7.0]), and the reaction mixture was incubated statically at 37°C until the sample turned yellow. At this point, 200 μl of 1 M NaCO_3_ was added to stop the reaction, and the samples were centrifuged at 21,130 × *g* for 30 s to remove any remaining cellular debris. The sample was transferred to a cuvette, and absorbance was measured at 420 nm. A Bradford assay was performed using protein assay dye reagent (Bio-Rad, Hercules, CA) to determine total protein content of all samples. Modified Miller unit values were calculated using the protein concentrations, and the data are reported on a per-milligram-of-protein basis (24).

### Eukaryotic cell culture and invasion.

HNEC cell invasion assays using RPMI 2650 cells were performed as outlined previously (23) with a number of modifications detailed below. RPMI 2650 cells were purchased from ATCC and routinely cultured in Eagle’s minimum essential medium (EMEM) supplemented with 10% heat-inactivated FBS and 1% of penicillin/streptomycin. Cells were grown in 75 cm^2^ cell culture flasks in a 37°C incubator supplemented with 5% CO_2_ and were subcultured according to ATCC protocols. Forty-eight h prior to infection with S. aureus, RPMI 2650 cells were seeded at a density of 2 × 10^5^ cells per well in 24-well plates. Once seeded in 24-well plates, cells to be infected at 34°C were maintained at 34°C in 5% CO_2_, while cells to be infected at 37°C were maintained at 37°C in 5% CO_2_. To prepare inocula, wild-type S. aureus was grown at either 34°C or 37°C in TSB overnight. Cultures were washed twice in phosphate-buffered saline (PBS) and resuspended in EMEM at a concentration of 4 × 10^6^ CFU/ml. To initiate the infection, 500 μl of inocula (corresponding to 2 × 10^6^ CFU) was added to each well to give a multiplicity of infection (MOI) of 10. Bacteria grown at 34°C were used to infect RPMI 2650 cells in 24-well plates maintained at 34°C, while bacteria grown at 37°C were used to infect RPMI 2650 cells maintained at 37°C. Plates were centrifuged at 1,000 rpm for 10 min at room temperature and placed at 34°C or 37°C for 60 min to allow internalization of the bacteria. After the 60 min incubation, the medium was aspirated, and the wells were washed once with prewarmed PBS. To kill extracellular bacteria, 500 μl of EMEM containing 30 μg/ml of gentamicin was added. Cells were incubated at 34°C or 37°C for 60 min. The end of this 60 min incubation was designated time zero (*T* = 0). At time zero, the medium containing 30 μg/ml of gentamicin was aspirated and replaced with EMEM containing 5 μg/ml of gentamicin for the remainder of the infection. To determine the number of intracellular bacteria at each time point, the medium was aspirated, cells were washed 2× with prewarmed PBS, and RPMI 2650 cells were lysed by adding 500 μl of 0.5% Triton X-100 in PBS to each well. The lysates were serially diluted and plated on TSA to determine bacterial load. To determine the effect of cell invasion on RPMI 2650 cell viability, an MTT assay was performed at each time point (at both temperatures) on infected and uninfected RPMI 2650 cells using the Vybrant MTT cell proliferation assay kit (ThermoFisher).

### Ethical statement.

Whole human blood was isolated from donors in agreement with the Ohio University Institutional Review Board (identification code, 17-X-79; date of approval, 22 February 2019).

### Data analysis.

The mass spectrometry proteomics data have been deposited to the ProteomeXchange Consortium via the PRIDE (50) partner repository with the data set identifier PXD023039. RNAseq data are deposited in the Gene Expression Omnibus (GEO) database under the accession number GSE162697.
